# Alcohol consumption and gastric cancer risk: a meta-analysis of prospective cohort studies

**DOI:** 10.18632/oncotarget.19177

**Published:** 2017-07-12

**Authors:** Xue Han, Li Xiao, Yao Yu, Yu Chen, Hai-Hua Shu

**Affiliations:** ^1^ Department of Anesthesiology, Guangdong Second Provincial General Hospital, Guangzhou, China; ^2^ Department of Anesthesiology, First Affiliated Hospital, Sun Yat-sen University, Guangzhou, China; ^3^ Department of Anesthesiology, Second Affiliated Hospital of Dalian Medical University, Dalian, China

**Keywords:** alcohol consumption, meta-analysis, gastric cancer, dose-risk relation

## Abstract

We performed this meta-analysis to explore the precise quantification relationship between alcohol consumption and gastric cancer and to provide evidence for preventing gastric cancer. We searched PubMed, Embase, and Web of Science for articles published up to December 2016, and identified 23 cohort studies that included a total population of 5,886,792 subjects. We derived meta-analytic estimates using random-effects models, taking into account correlations between estimates. We also investigated the dose–response relationship between gastric cancer risk and alcohol consumption. We found that alcohol consumption increased gastric cancer risk, where the summary risk ratio was 1.17 (95% confidence interval (CI): 1.00–1.34; *I*^2^ = 79.6%, *p* < 0.05. The dose–response analysis showed that every 10 g/d increment in alcohol consumption was associated with 7% increased gastric cancer risk (95% CI 1.02–1.12; *I*^2^ = 28.9%, *p* = 0.002). This meta-analysis provides evidence that alcohol consumption is an important risk factor of the incidence of gastric cancer.

## INTRODUCTION

Although the death rate of gastric cancer has declined for several decades, epidemiological data from the American Association of Cancer suggest that gastric cancer is the fourth and fifth most common cancer in men and women, respectively, and it remains a major public health problem worldwide [1, 2]. As the mortality rate of gastric cancer remains high, a specific prevention strategy is urgently needed.

The occurrence of gastric cancer involves a multi-factorial (*Helicobacter pylori* infection, dietary factors, smoking, family history of cancer), multi-stage developmental process; the exact cause of the disease is still not fully understood [3, 4]. Lifestyle habits and diet may play key roles in the etiology of gastric cancer [5, 6]. Many studies have shown that alcohol consumption affects gastric cancer cell proliferation and cell cycle distribution and apoptosis [7–9]. The results of a meta-analyses published in 2012 [10] suggested a slight association between alcohol consumption and gastric cancer risk [11–24], although the authors did not evaluate the quality of the articles or classify them. In recent decades, numerous prospective cohort studies have investigated the association between alcohol consumption and gastric cancer [25–33]. The results from these studies were inconsistent; therefore, a comprehensive meta-analysis is necessary.

To explore the relationship between gastric cancer incidence and alcohol consumption and to provide a scientific basis for gastric cancer prevention, we conducted a meta-analysis of the evidence across all prospective cohort studies published in 1987–2016.

## RESULTS

**Figure 1:**
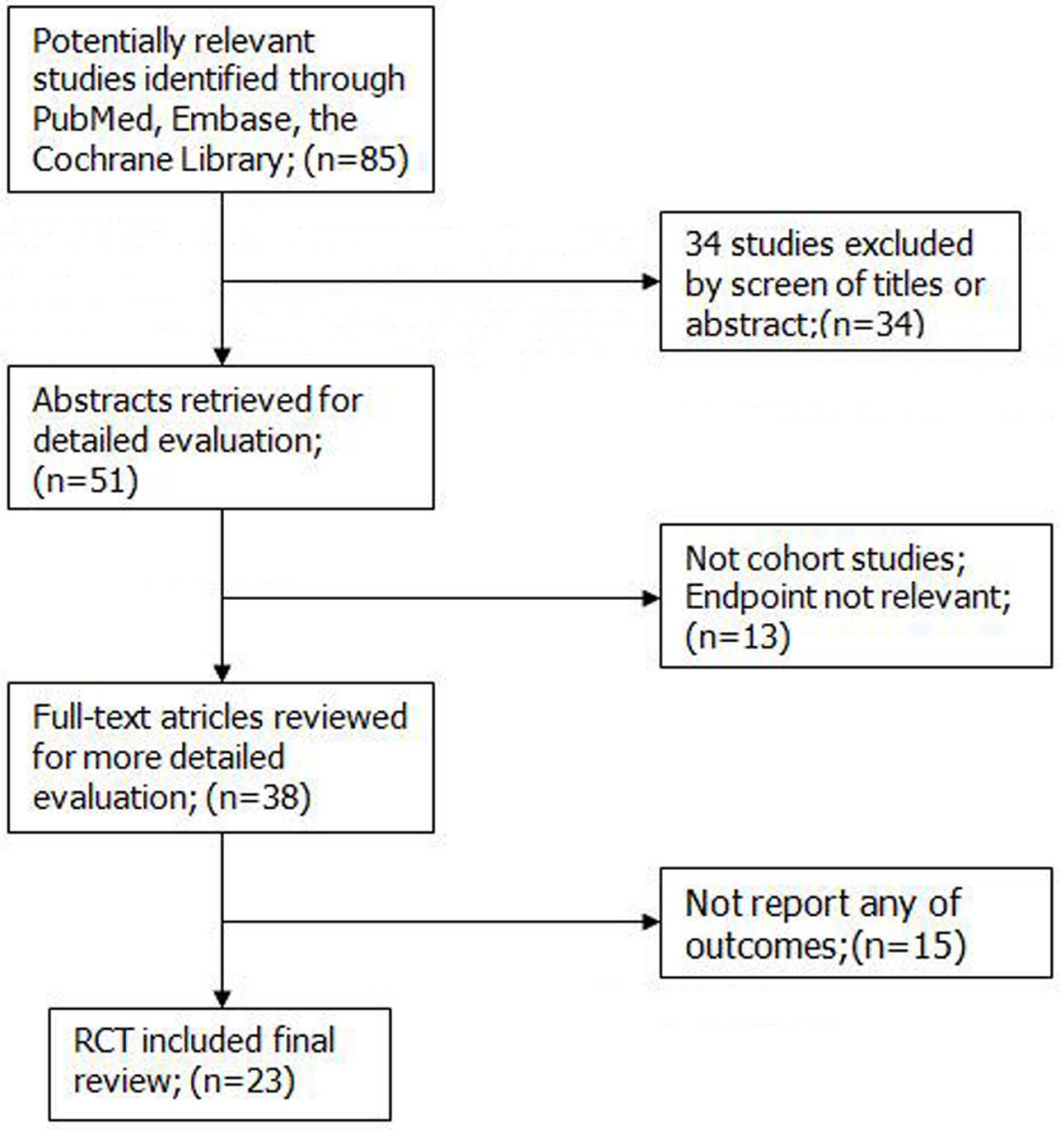
Search flow diagram for studies included in the meta-analysis RCT, randomized controlled trials.

Figure 1 depicts the study selection process; we initially reviewed 85 potentially relevant records, and an eventual 23 articles, involving 5,886,792 subjects, that met the inclusion criteria were included in the meta-analysis (Supplementary Table 1). We subsequently excluded 62 studies because they used combined intervention, were duplicate reports or studies, or their endpoints were not relevant. Among the 23 included articles, five had been conducted in Japan, four had been conducted in Korea, and three had been conducted in the USA and other countries (Brazil, Lithuania, the Netherlands, Norway, Denmark). Fourteen and nine studies had an impact factor of > 3 and ≤ 3, respectively. Seven, 13, and three studies had a NOS (Newcastle–Ottawa Quality Assessment Scale) score of 8, 7, and 6, respectively. We used the risk estimate that was the most representative of the most commonly consumed alcohol.

**Figure 2:**
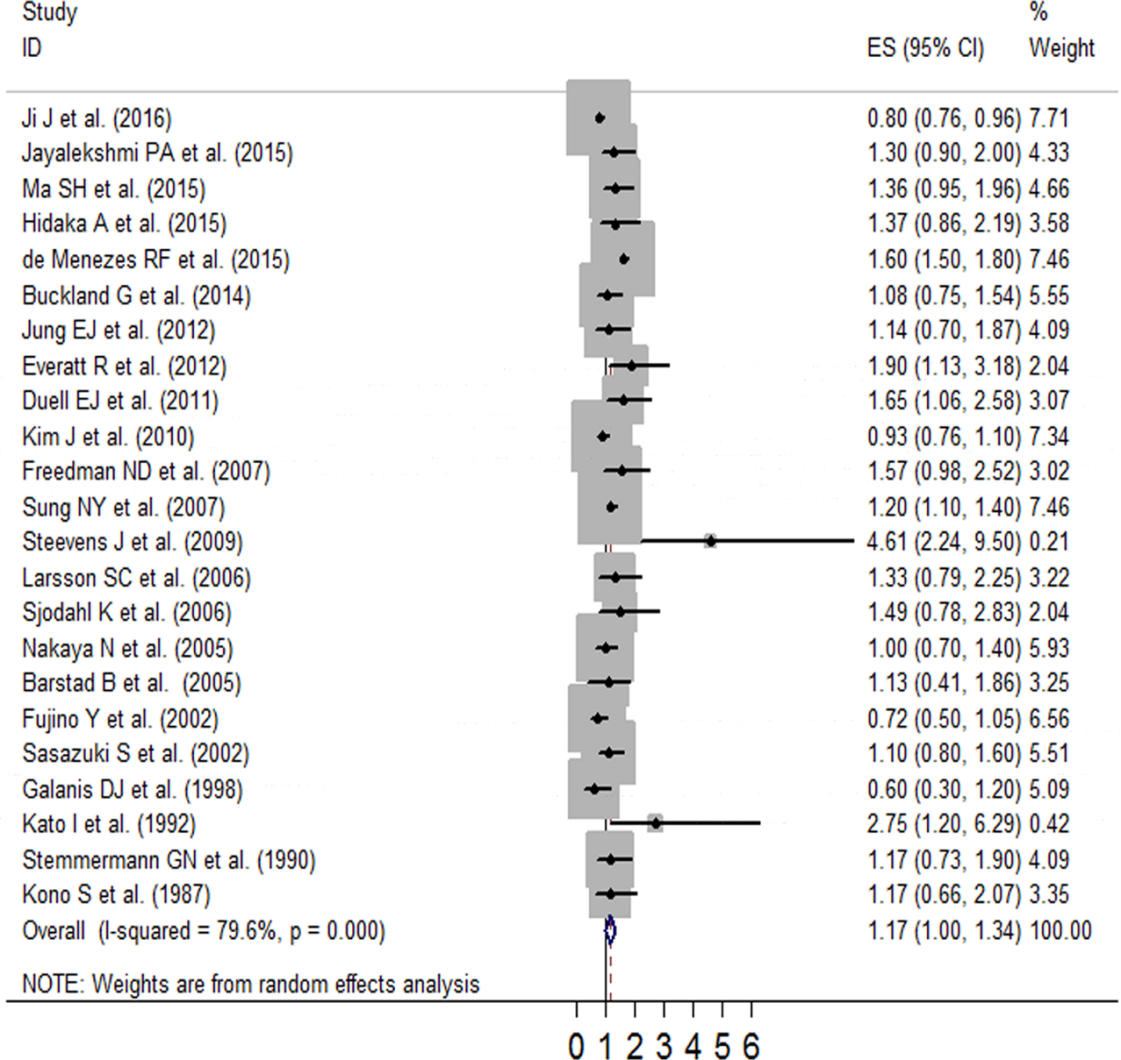
Forest plot of RR (with 95% CI) examining the association between alcohol intake and gastric cancer risk in a random-effects model

**Figure 3:**
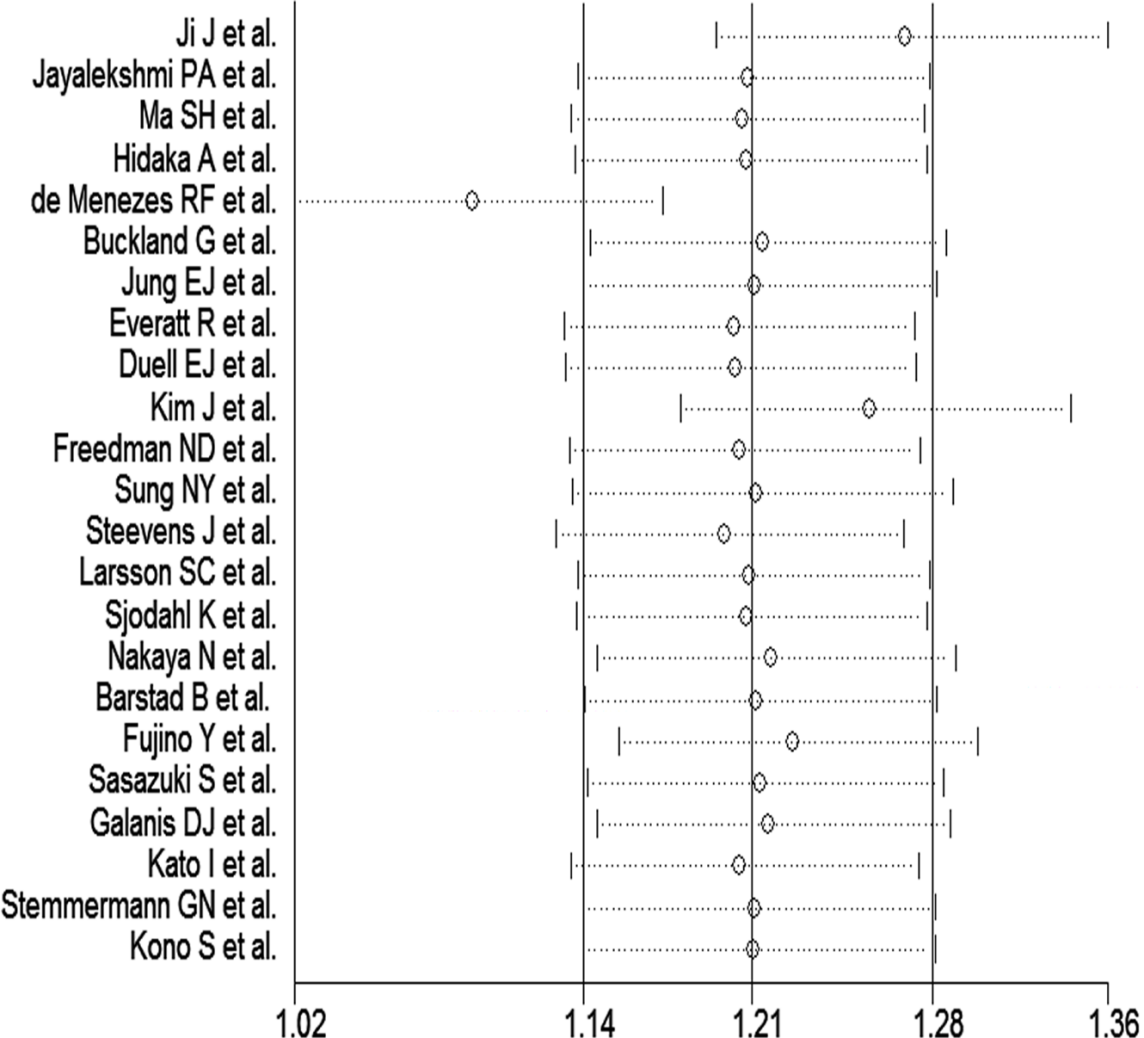
Sensitivity analysis of alcohol consumption and gastric cancer risk

**Figure 4:**
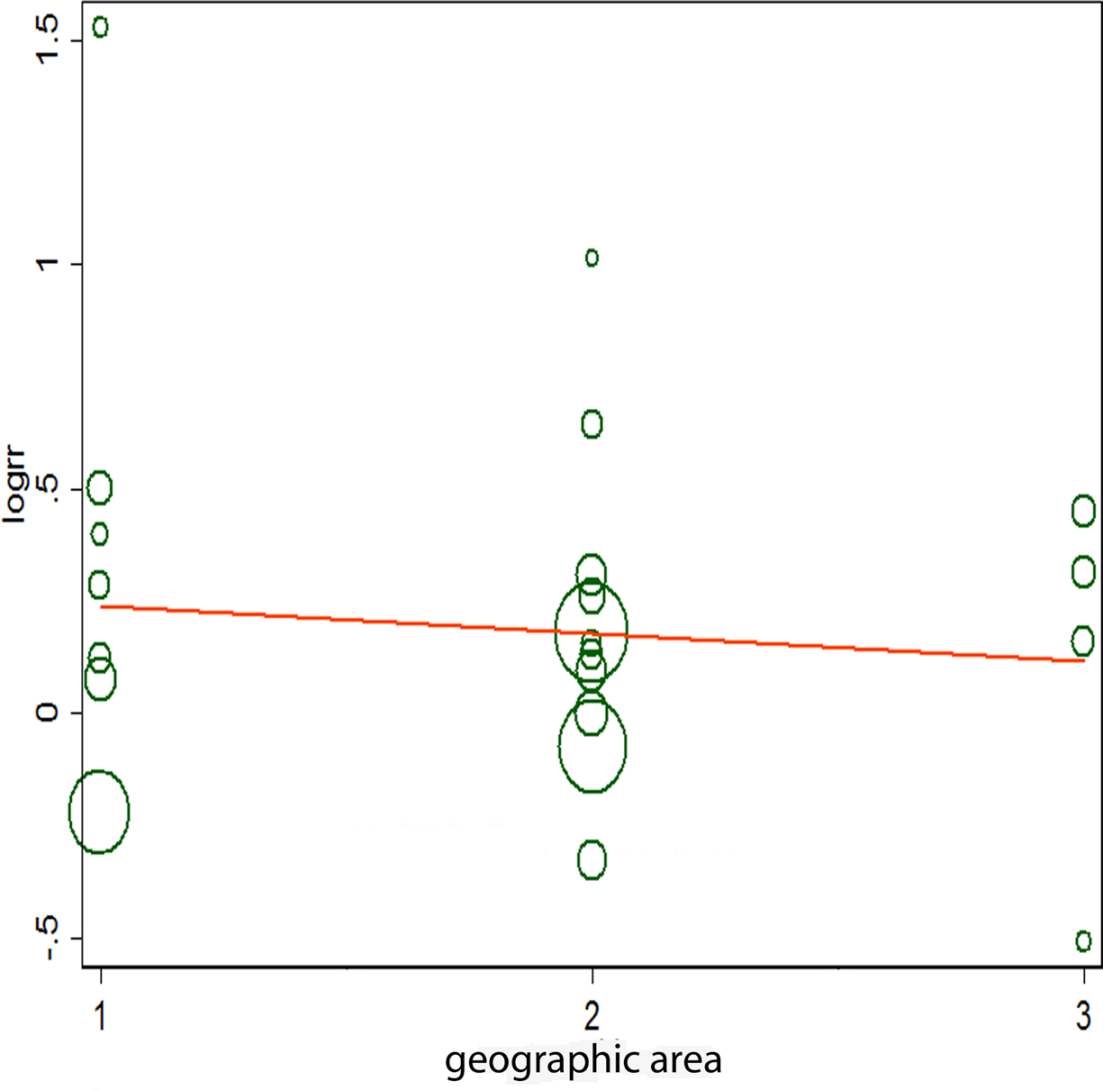
Meta-regulation of alcohol consumption and gastric cancer risk 1 represents Europe, 2 represents Asia, and 3 represents America.

We generated forest plots for alcohol consumption and gastric cancer. The results from the 23 studies were inconsistent: two studies reported that alcohol consumption was associated with significantly reduced gastric cancer risk, and six studies reported no association; however, 15 studies reported significantly increased gastric cancer risk. Analysis of the 23 studies yielded a combined risk estimate of 1.17 (95% confidence interval (CI), 1.00–1.34; *p* < 0.05) with a heterogeneity value (*I*^*2*^) of 79.6% (Figure 2). We conducted sensitivity analysis (Figure 3) and meta-regulation testing (Figure 4). As the figure shows, the results showed that the omission of any study did not alter the observed effect, which revealed that the publication dates were similar. Geographic area was associated with ∼53.7% heterogeneity reduction across the studies.

As the studies differed in terms of geographic area (Asia, America, Europe), sex (female or male), impact factor (> 3 or ≤ 3), and NOS score (6/7/8), we conducted subgroup analyses to determine the effect of these factors on our analyses (Table 1). We obtained a statistically significant protective effect of alcohol consumption (relative risk [RR]: 0.85; 95% CI: 0.76–0.95) in Europe, and a statistically significant harmful effect of alcohol consumption in the studies that adjusted for age, education level, smoking status, and body mass index (BMI), studies with NOS scores of 8, impact factor > 3, and in men and in Americans.

**Table 1:** Subgroup analyses of gastric cancer and alcohol consumption

Group	No. of studies	RR (95% CI)	***P***_heterogeneity_	*I*^*2*^ (%)
**Adjustment**				
**Age**				
Yes	20	1.06 (1.0, 1.12)	0	82
**Sex**				
Yes	11	0.9 (0.82, 0.98)	0.006	59.1
**Education**				
Yes	11	1.39 (1.27, 1.50)	0.001	65.7
**Smoking status**				
Yes	16	1.25 (1.16, 1.34 )	0	71.8
**Family history of gastric cancer**				
Yes	3	0.96 (0.80, 1.13)	0.176	42.5
**BMI**				
Yes	11	1.13 (1.03, 1.23)	0.136	32.9
**NOS**				
6	3	0.81 (0.72, 0.91)	0.410	0
7	13	1.09 (0.91,1.21)	0.097	35.7
8	7	1.27 (1.00, 1.54)	0	78.3
**IF**				
> 3	14	1.10 (1.01, 1.19)	0.008	35.8
≤ 3	9	1.04 (0.96, 1.11)	0	90.8
**Sex**				
M	7	1.18 (1.06, 1.30)	0.768	0
W	1	1.13 (0.79, 2.25)	0	0
M+W	15	1.02 (0.96, 1.09)	0	85.9
**Geographic region**				
Europe	7	0.85 (0.76, 0.95)	0.026	58.2
America	5	1.48 (1.35, 1.81)	0.001	78.2
Asia	11	1.06 (0.97, 1.16)	0.053	44.9

IF = impact factor; No. = number; RR = relative risk; CI = confidence interval; M = men; W = women; BMI = body mass index; NOS: Newcastle–Ottawa Quality Assessment Scale.

**Figure 5:**
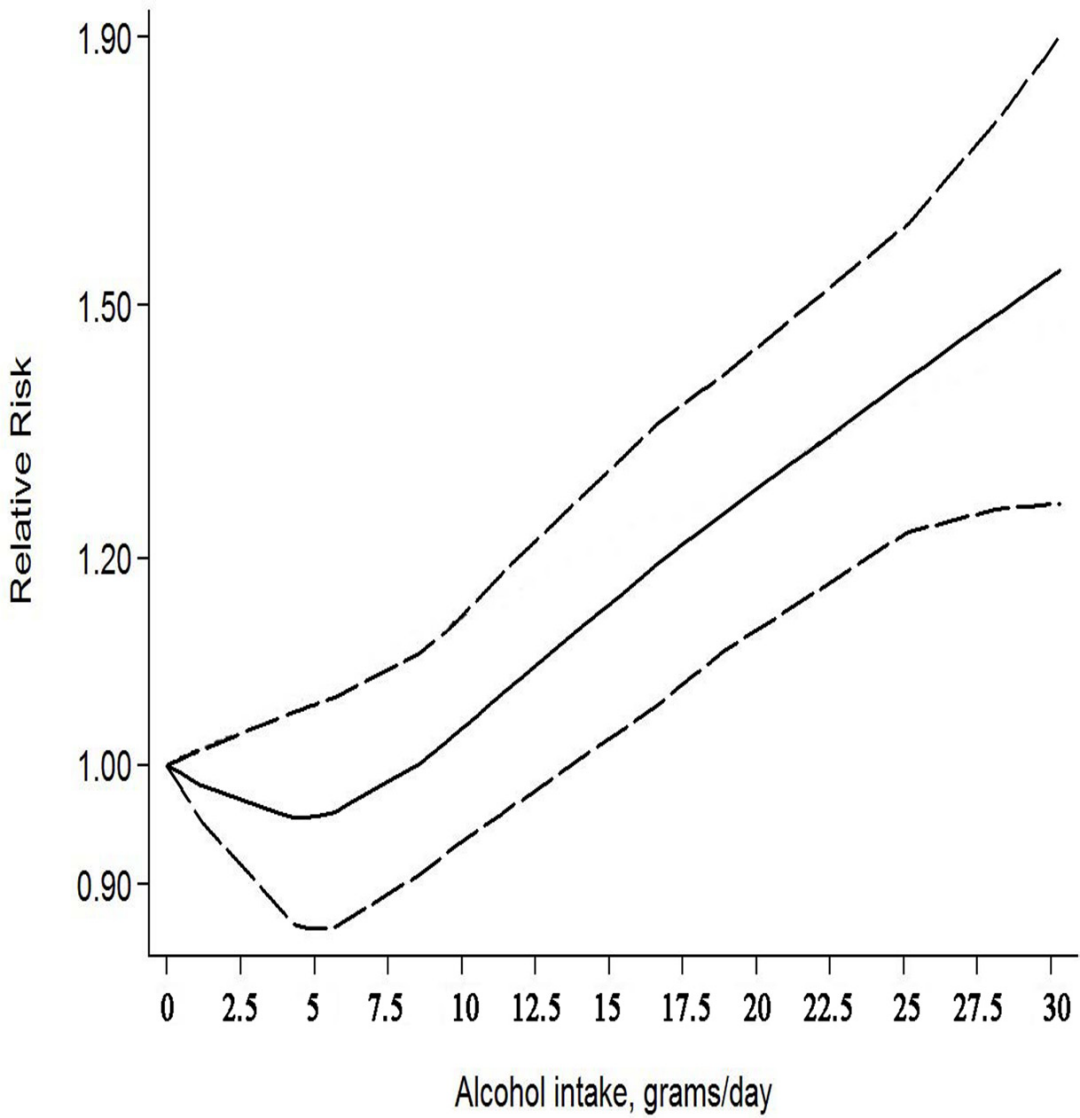
The dose–response relationship between alcohol consumption and gastric cancer risk

Dose–response analysis was performed using the available data (Figure 5). Overall, an increment of 10 g/d alcohol intake was associated with a significant risk increase of 7% (RR = 1.07, 95% CI: 1.02–1.12). No evidence of heterogeneity was observed for exposure (*p* > 0.5).

**Figure 6:**
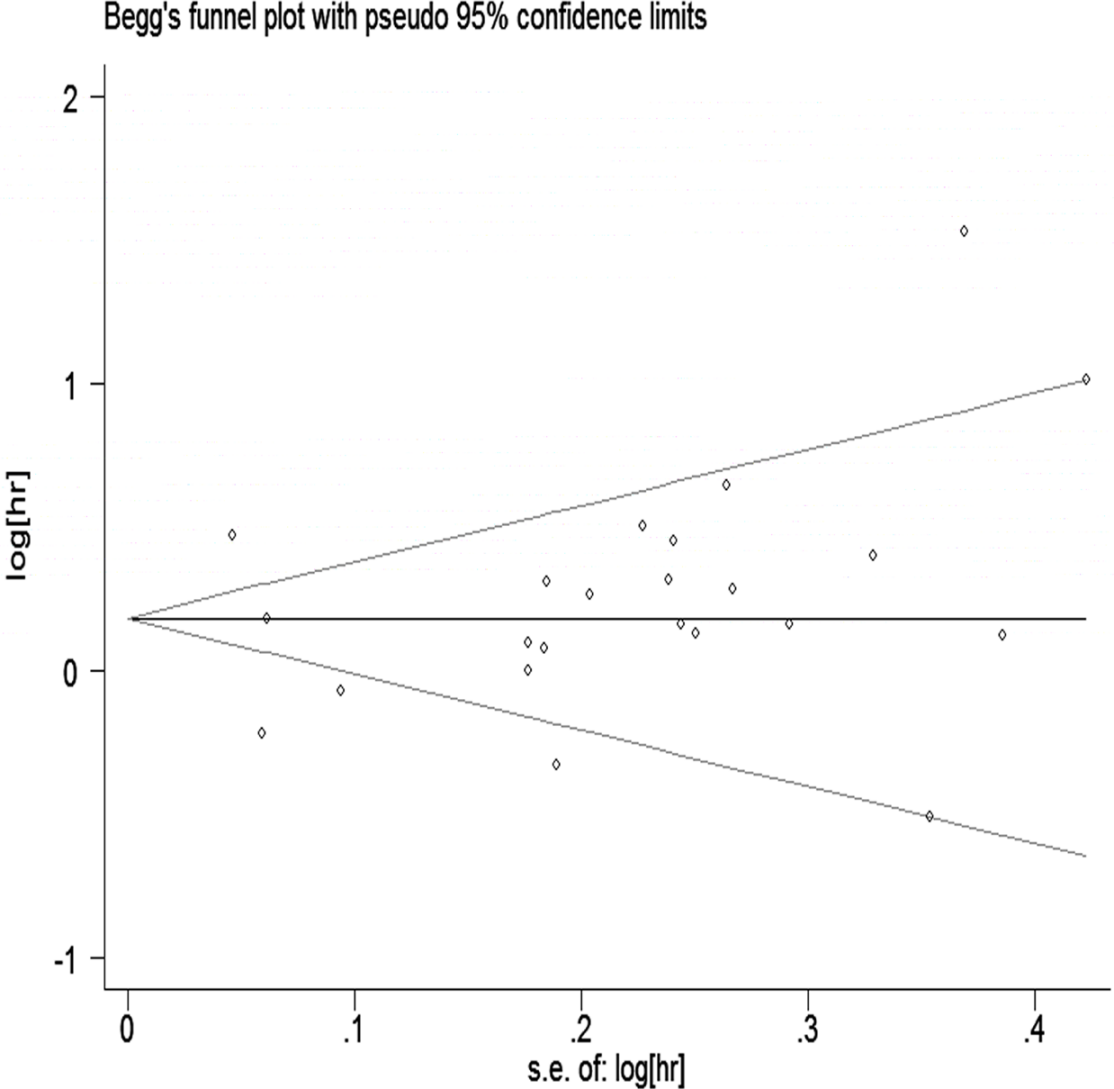
Egger’s funnel plot assessing publication bias among the studies

**Figure 7:**
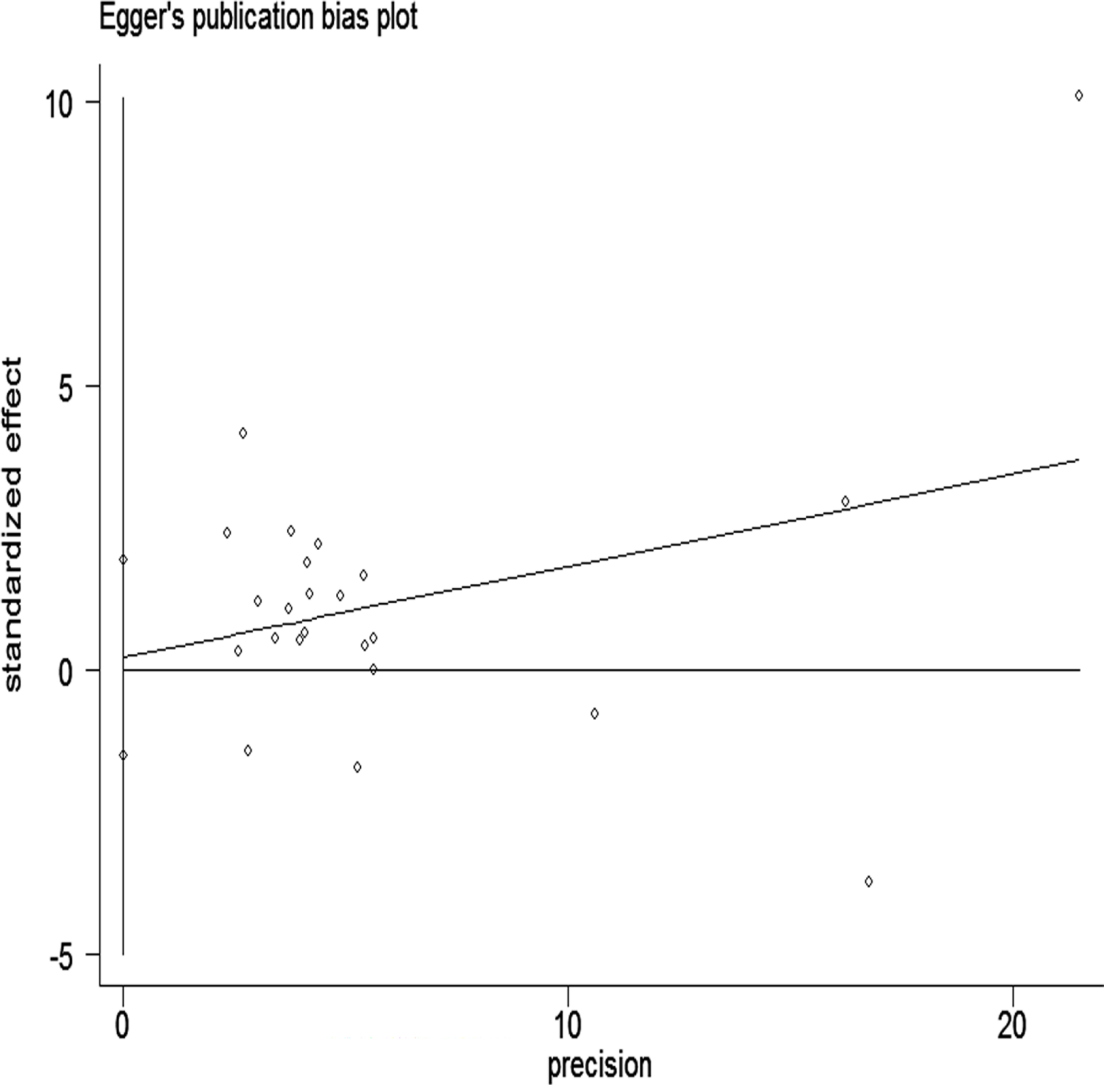
Begg’s funnel plot with pseudo-95% CI assessing publication bias among the studies

The Egger and Begg regression models [34] revealed no evidence of publication bias (Figures 6 and 7) with regard to the consumption of alcohol in relation to gastric cancer risk. The Egger funnel plot and Egger linear regression test yielded *p* > 0.05 and *p* > 0.05, respectively.

## DISCUSSION

Meta-analysis is a method of summarizing the results of the same research purpose and comprehensively evaluating its combined effect. It can be objective, systematic, comprehensive, qualitative, and quantitative statistical analysis [35]. Considering consistency, meta-analysis can be used to combine all available information, the range of total odds ratio (OR) values obtained by convergence, resolve inconsistent or even mutually contradictory original research results. It has functions that improve estimates of effect, construct a general review method for omitting inadequate study conclusions, and reinforce the effectiveness of statistical results to yield more comprehensive and reliable study results that are more representative of the general population.

The occurrence of gastric cancer is closely related not only to dietary factors, but also some non-dietary factors, which play an important role [36]. We used meta-analysis to evaluate the causal connections in gastric cancer etiology in terms of strength and specificity. We performed a comprehensive quantitative analysis spanning 30 years and 23 different occasions, locations, and high-quality studies. We determined whether the authors discussed the relationship between gastric cancer and alcohol consumption and evaluated the relationship between strength and contact where the alcohol risk factor for gastric cancer was 1.06 (RR: 1–1.12). We found that alcohol consumption can increase gastric cancer risk. In our dose–response analysis, every 10 g/d increment of alcohol consumption was associated with 7% increased gastric cancer risk, meaning that high-dose alcohol drinkers had higher gastric cancer risk than low-dose alcohol drinkers. However, the specific mechanism between alcohol consumption and gastric cancer risk is not clear. Several researchers have attempted to explain the relationship between gastric cancer occurrence and alcohol consumption. For example, alcohol can significantly increase the carcinogenicity of *N*-nitroso compounds [37, 38]. In drinkers of liquor in particular, which has a high alcohol content, the damage stimulates the gastric mucosa, and the resultant mucosal changes give rise to gastric cancer cells. *H. pylori* is another risk factor of gastric cancer [39, 40]; therefore, adequate adjustment for *H. pylori* is essential. However, only one study reported the RR for alcohol consumption and gastric cancer adjusted for *H. pylori* [27].

The limitations of the present meta-analysis should be noted. Only studies written in English were included; studies that were not written in English would have been overlooked. In our meta-analysis, there was heterogeneity across the studies, and regulation testing showed that geographical area was associated with ∼53.7% reduction in heterogeneity across the studies. We did not have information on wine variety classification; as wine varieties might differ between regions, the results would also have differed. Furthermore, heterogeneity among studies was allowed to account for the use of the random-effects model. Meta-analysis is an observational study, and bias in the design, data collection, and statistical analysis of a meta-analysis is unavoidable; accordingly, the quality of the data is used to evaluate and decide if here will be bias.

Our meta-analysis had several strengths. To improve the statistical power, we included several studies, all of which were prospective cohort studies. This design minimizes selection and recall bias.

In conclusion, our meta-analysis reveals a negative association between alcohol consumption and gastric cancer risk, with an RR value of 1.17 (95% CI: 1.00–1.34, *p* < 0.05 and an *I*^*2*^ value of 79.6%. Due to the limitations mentioned above, more high-quality cohort studies are required to strengthen the findings.

## MATERIALS AND METHODS

### Search strategy

We updated the systematic literature review published in 2012 [10]. We used the PubMed (http://www.ncbi.nlm.nih.gov/pubmed/), Embase (http://www.embase.com/), Web of Science (http://wokinfo.com/), and Cochrane Library (http://www.thecochranelibrary.com/) databases to identify articles using the terms “alcohol beverages/consumption/drinking” and “gastric/stomach cancer/neoplasms/tumor/malignancy”. In addition, we reviewed the reference lists of the 23 included studies to identify additional studies. The language of the studies was limited to English, and we did not search for unpublished studies. In addition to the reference lists of the included studies, we also searched those listed in the Meta-analysis Of Observational Studies in Epidemiology guidelines [41, 42]. We followed standard criteria for conducting and reporting the meta-analysis [43].

### Study selection

We included prospective cohorts of alcohol consumption and gastric cancer incidence. The inclusion criteria were: (1) a prospective cohort design; (2) investigation of the association between alcohol consumption and gastric cancer incidence; (3) reported the OR or RR with 95% CI; and (4) prospective cohort studies. For the dose–response analysis, a quantitative measure of intake had to be provided. When there were several publications from the same study, we selected the publication with the largest number of cases and the longest study period.

### Data extraction and quality assessment

From each study, we extracted the first author’s last name, year of publication, country in which the study was conducted, age, duration, number of cases, exposure range, adjusted OR, RR, or hazard ratio (HR) with the 95% CI and adjustments, NOS score, and impact factor (Supplementary Table 1). The RR was used as the common measure of association across studies, and the OR and HR were directly considered the RR. We used *Q* and *I*^*2*^ statistics to estimate heterogeneity among the studies; disagreements were resolved by discussion. Two authors assessed the methodological quality of the included studies independently using the NOS.

### Statistical methods

The association between alcohol consumption and gastric cancer risk was assessed using the RR. We used *Q* (*p* ≤ 0.10) and *I*^*2*^ statistics to examine heterogeneity across the studies and used the random-effects model when substantial heterogeneity was detected [44]. Subgroup analyses were conducted on adjustment for age, sex, education level, smoking status, family history of gastric cancer, BMI, NOS score, impact factor, sex distribution of the study population, and geographic region. Additionally, we investigated the effect of a single study on the overall risk estimate (Figure 3). This allowed us to determine whether a single study could have affected the results significantly.

Data analyses were performed with STATA version 13.0; *p* < 0.05 was considered statistically significant. We used Egger’s linear regression and Begg’s rank correlation to evaluate potential publication bias.

## SUPPLEMENTARY MATERIALS TABLE





## References

[1] Torre LA, Bray F, Siegel RL, Ferlay J, Lortet-Tieulent J, Jemal A (2015). Global cancer statistics, 2012. CA Cancer J Clin.

[2] Soerjomataram I, Lortet-Tieulent J, Parkin DM, Ferlay J, Mathers C, Forman D, Bray F (2012). Global burden of cancer in 2008: a systematic analysis of disability-adjusted life-years in 12 world regions. Lancet.

[3] Eom BW, Kim YW, Nam BH, Ryu KW, Jeong HY, Park YK, Lee YJ, Yang HK, Yu W, Yook JH, Song GA, Youn SJ, Kim HU (2016). The Korean Gastric Cancer Cohort Study: Study Protocol and Brief Results of a Large-Scale Prospective Cohort Study. J Gastric Cancer.

[4] Baroudi O, Benammar-Elgaaied A (2016). Involvement of genetic factors and lifestyle on the occurrence of colorectal and gastric cancer. Crit Rev Oncol Hematol.

[5] Navarro Silvera SA, Mayne ST, Gammon MD, Vaughan TL, Chow WH, Dubin JA, Dubrow R, Stanford JL, West AB, Rotterdam H, Blot WJ, Risch HA (2014). Diet and lifestyle factors and risk of subtypes of esophageal and gastric cancers: classification tree analysis. Ann Epidemiol.

[6] Larghi A, Panic N, Capurso G, Leoncini E, Arzani D, Salvia R, Del Chiaro M, Frulloni L, Arcidiacono PG, Zerbi A, Manta R, Fabbri C, Ventrucci M (2013). Prevalence and risk factors of extrapancreatic malignancies in a large cohort of patients with intraductal papillary mucinous neoplasm (IPMN) of the pancreas. Ann Oncol.

[7] Cheng XJ, Lin JC, Tu SP (2016). Etiology and Prevention of Gastric Cancer. Gastrointest Tumors.

[8] An J, Zhao J, Zhang X, Ding R, Geng T, Feng T, Jin T (2015). Impact of multiple Alcohol Dehydrogenase gene polymorphisms on risk of laryngeal, esophageal, gastric and colorectal cancers in Chinese Han population. Am J Cancer Res.

[9] Jelski W, Chrostek L, Zalewski B, Szmitkowski M (2008). Alcohol dehydrogenase (ADH) isoenzymes and aldehyde dehydrogenase (ALDH) activity in the sera of patients with gastric cancer. Dig Dis Sci.

[10] Tramacere I, Negri E, Pelucchi C, Bagnardi V, Rota M, Scotti L, Islami F, Corrao G, La Vecchia C, Boffetta P (2012). A meta-analysis on alcohol drinking and gastric cancer risk. Ann Oncol.

[11] Steevens J, Schouten LJ, Goldbohm RA, van den Brandt PA (2010). Alcohol consumption, cigarette smoking and risk of subtypes of oesophageal and gastric cancer: a prospective cohort study. Gut.

[12] Kim J, Park S, Nam BH (2010). Gastric cancer and salt preference: a population-based cohort study in Korea. Am J Clin Nutr.

[13] Sung NY, Choi KS, Park EC, Park K, Lee SY, Lee AK, Choi IJ, Jung KW, Won YJ, Shin HR (2007). Smoking, alcohol and gastric cancer risk in Korean men: the National Health Insurance Corporation Study. Br J Cancer.

[14] Sjodahl K, Lu Y, Nilsen TI, Ye W, Hveem K, Vatten L, Lagergren J (2007). Smoking and alcohol drinking in relation to risk of gastric cancer: a population-based, prospective cohort study. Int J Cancer.

[15] Larsson SC, Giovannucci E, Wolk A (2007). Alcoholic beverage consumption and gastric cancer risk: a prospective population-based study in women. Int J Cancer.

[16] Freedman ND, Abnet CC, Leitzmann MF, Mouw T, Subar AF, Hollenbeck AR, Schatzkin A (2007). A prospective study of tobacco, alcohol, and the risk of esophageal and gastric cancer subtypes. Am J Epidemiol.

[17] Nakaya N, Tsubono Y, Kuriyama S, Hozawa A, Shimazu T, Kurashima K, Fukudo S, Shibuya D, Tsuji I (2005). Alcohol consumption and the risk of cancer in Japanese men: the Miyagi cohort study. Eur J Cancer Prev.

[18] Barstad B, Sorensen TI, Tjonneland A, Johansen D, Becker U, Andersen IB, Gronbaek M (2005). Intake of wine, beer and spirits and risk of gastric cancer. Eur J Cancer Prev.

[19] Sasazuki S, Sasaki S, Tsugane S, Japan Public Health Center Study Group (2002). Cigarette smoking, alcohol consumption and subsequent gastric cancer risk by subsite and histologic type. Int J Cancer.

[20] Fujino Y, Tamakoshi A, Ohno Y, Mizoue T, Tokui N, Yoshimura T (2002). Risk JSGJCCSfEoC. Prospective study of educational background and stomach cancer in Japan. Prev Med.

[21] Galanis DJ, Kolonel LN, Lee J, Nomura A (1998). Intakes of selected foods and beverages and the incidence of gastric cancer among the Japanese residents of Hawaii: a prospective study. Int J Epidemiol.

[22] Kato I, Tominaga S, Matsumoto K (1992). A prospective study of stomach cancer among a rural Japanese population: a 6-year survey. Jpn J Cancer Res.

[23] Stemmermann GN, Nomura AM, Chyou PH, Yoshizawa C (1990). Prospective study of alcohol intake and large bowel cancer. Dig Dis Sci.

[24] Kono S, Ikeda M, Tokudome S, Nishizumi M, Kuratsune M (1987). Cigarette smoking, alcohol and cancer mortality: a cohort study of male Japanese physicians. Jpn J Cancer Res.

[25] Ji J, Sundquist J, Sundquist K (2016). Associations of alcohol use disorders with esophageal and gastric cancers: a population-based study in Sweden. Eur J Cancer Prev.

[26] Jayalekshmi PA, Hassani S, Nandakumar A, Koriyama C, Sebastian P, Akiba S (2015). Gastric cancer risk in relation to tobacco use and alcohol drinking in Kerala, India—Karunagappally cohort study. World J Gastroenterol.

[27] Hidaka A, Sasazuki S, Matsuo K, Ito H, Sawada N, Shimazu T, Yamaji T, Iwasaki M, Inoue M, Tsugane S, Group JS (2015). Genetic polymorphisms of ADH1B, ADH1C, ALDH2, alcohol consumption, and the risk of gastric cancer: the Japan Public Health Center-based prospective study. Carcinogenesis.

[28] Hashibe M, Galeone C, Buys SS, Gren L, Boffetta P, Zhang ZF, La Vecchia C (2015). Coffee, tea, caffeine intake, and the risk of cancer in the PLCO cohort. Br J Cancer.

[29] de Menezes RF, Bergmann A, de Aguiar SS, Thuler LC (2015). Alcohol consumption and the risk of cancer in Brazil: A study involving 203,506 cancer patients. Alcohol.

[30] Buckland G, Travier N, Huerta JM, Bueno-de-Mesquita HB, Siersema PD, Skeie G, Weiderpass E, Engeset D, Ericson U, Ohlsson B, Agudo A, Romieu I, Ferrari P (2015). Healthy lifestyle index and risk of gastric adenocarcinoma in the EPIC cohort study. Int J Cancer.

[31] Jung EJ, Shin A, Park SK, Ma SH, Cho IS, Park B, Lee EH, Chang SH, Shin HR, Kang D, Yoo KY (2012). Alcohol consumption and mortality in the Korean Multi-Center Cancer Cohort Study. J Prev Med Public Health.

[32] Everatt R, Tamosiunas A, Kuzmickiene I, Virviciute D, Radisauskas R, Reklaitiene R, Milinaviciene E (2012). Alcohol consumption and risk of gastric cancer: a cohort study of men in Kaunas, Lithuania, with up to 30 years follow-up. BMC Cancer.

[33] Duell EJ, Travier N, Lujan-Barroso L, Clavel-Chapelon F, Boutron-Ruault MC, Morois S, Palli D, Krogh V, Panico S, Tumino R, Sacerdote C, Quiros JR, Sanchez-Cantalejo E (2011). Alcohol consumption and gastric cancer risk in the European Prospective Investigation into Cancer and Nutrition (EPIC) cohort. Am J Clin Nutr.

[34] Begg CB, Mazumdar M (1994). Operating characteristics of a rank correlation test for publication bias. Biometrics.

[35] Feichtinger J, Aldeailej I, Anderson R, Almutairi M, Almatrafi A, Alsiwiehri N, Griffiths K, Stuart N, Wakeman JA, Larcombe L, McFarlane RJ (2012). Meta-analysis of clinical data using human meiotic genes identifies a novel cohort of highly restricted cancer-specific marker genes. Oncotarget.

[36] Fang X, Wei J, He X, An P, Wang H, Jiang L, Shao D, Liang H, Li Y, Wang F, Min J (2015). Landscape of dietary factors associated with risk of gastric cancer: A systematic review and dose-response meta-analysis of prospective cohort studies. Eur J Cancer.

[37] Xu L, Qu YH, Chu XD, Wang R, Nelson HH, Gao YT, Yuan JM (2015). Urinary levels of N-nitroso compounds in relation to risk of gastric cancer: findings from the shanghai cohort study. PLoS One.

[38] Heo SH, Jeong ES, Lee KS, Seo JH, Jeong DG, Won YS, Kwon HJ, Kim HC, Kim DY, Choi YK (2013). Canonical Wnt signaling pathway plays an essential role in N-methyl-N-nitrosurea induced gastric tumorigenesis of mice. J Vet Med Sci.

[39] Liu KS, Wong IO, Leung WK (2016). Helicobacter pylori associated gastric intestinal metaplasia: Treatment and surveillance. World J Gastroenterol.

[40] Song ZQ, Zhou LY (2015). Helicobacter Pylori and Gastric Cancer: Clinical Aspects. Chin Med J (Engl).

[41] Moon JH (2016). Endocrine Risk Factors for Cognitive Impairment. Endocrinol Metab (Seoul).

[42] Huncharek M, Muscat J, Kupelnick B (2009). Colorectal cancer risk and dietary intake of calcium, vitamin D, and dairy products: a meta-analysis of 26,335 cases from 60 observational studies. Nutr Cancer.

[43] Tao KM, Li XQ, Zhou QH, Moher D, Ling CQ, Yu WF (2011). From QUOROM to PRISMA: a survey of high-impact medical journals’ instructions to authors and a review of systematic reviews in anesthesia literature. PLoS One.

[44] DerSimonian R, Laird N (2015). Meta-analysis in clinical trials revisited. Contemp Clin Trials.

