# Supplementary material for: Alcohol consumption and gastric cancer risk: a meta-analysis of prospective cohort studies
Source: Oncotarget. 2017 Jul 12;8(47):83237–45. doi: 10.18632/oncotarget.19177 (PMC5669963; doi:10.18632/oncotarget.19177)
Supplement: Supplementary file 1 [file oncotarget-08-83237-s001.pdf]

Alcohol consumption and gastric cancer risk: a meta-analysis of prospective cohort studies

SUPPLEMENTARY MATERIALS

Supplementary Table 1: Cohort studies on the association between alcohol consumption and gastric cancer risk. See Supplementary_Table_1
