# Supplementary material for: Alcohol consumption and gastric cancer risk: a meta-analysis of prospective cohort studies
Source: Oncotarget. 2017 Jul 12;8(47):83237–45. doi: 10.18632/oncotarget.19177 (PMC5669963; doi:10.18632/oncotarget.19177)
Supplement: Supplementary file 2 [file oncotarget-08-83237-s002.DOCX]

**Supplementary Table 1: Cohort studies on the association between alcohol consumption and gastric cancer risk**

| Study | Year | | | Geographic area | Age (range, years) | Duration (years) | Population | Exposure range (g/d) | Adjusted RR  (95% CI) | Adjustment for covariates | NOS | IF |
| --- | --- | --- | --- | --- | --- | --- | --- | --- | --- | --- | --- | --- |
| Ji J et al. | 2016 | | Sweden | | 30-84 | 37 | 420,489 | High vs. low | 0.85 (0.76-0.96) | Sex, age, region, socioeconomic status, obesity, COPD. | 6 | <3 |
| Jayalekshmi PA et al. | 2015 | India | | | 30-84 | 19 | 65,553 | Current vs. never | 1.3  (0.9-2.0) | Age, calendar time, occupation, education level. | 7 | <3 |
| Ma SH et al. | 2015 | Korea | | | ≧20 | 11 | 18,863 | ≧55 vs. <25 (g per single occasion) | 1.36 (0.95–1.96) | Age, sex, BMI, education level, smoking status. | 7 | >3 |
| Hidaka A et al. | 2015 | Japan | | | 40-69 | 14 | 140,420 | ≧150 g/week vs. 0 | 1.57 (1.04-2.38) | Age, sex, area of residence, blood donation date and fasting time at blood donation, smoking status, BMI, total calorie and salt intake, family history of gastric cancer, *H. pylori* infection status, gastric atrophy, history of DM, *ALDH2* polymorphism. | 8 | >3 |
| De Menezes RF et al. | 2015 | Brazil | | | 18-100 | 9 | 203,506 | Current vs. never | 1.60 (1.50-1.80) | Sex, age, race, education level, marital status, smoking habit, region of residence, year of diagnosis. | 8 | <3 |
| Buckland G et al. | 2014 | European countries | | | 25-70 | 8 | 521,454 | High vs. low | 1.08 (0.75-1.54) | Physical activity; education level; total energy intake; stratification by sex, country, age at recruitment. | 7 | >3 |
| Jung EJ et al. | 2012 | Korea | | | ≧20 | 11 | 20,059 | Current vs. never | 1.14  (0.70- 1.87) | Age, sex, BMI, smoking habit, geographic area, education level. | 7 | <3 |
| Everatt R et al. | 2012 | Lithuania | | | 40-59 | 30 | 8,380 | ≧100.0 g/week vs. 0 | 1.90 (1.13-3.18) | Age, smoking, education level, BMI. | 7 | <3 |
| Duell EJ et al. | 2011 | European | | | 35-70 | 7 | 52,1457 | ≧ 60 g/d vs. 0 | 1.65 (1.06-2.58) | Age; sex; center; education level; smoking habit; fruit/nut/seed, vegetable, processed and red meat, total energy intake. | 8 | >3 |
| Kim J et al. | 2010 | Korea | | | 30–80 | 6-7 | 2,248,129 | Heavy vs. none | 0.93 (0.76-1.10) | Age, sex, BMI, smoking habit, alcohol consumption, physical activity, family history of cancer. | 7 | >3 |
| Steevens J et al. | 2009 | Dutch | | | 55–70 | 16.3 | 120,852 | ≧30 g/d vs. 0 | 4.61 (2.24-9.50) | Age; sex; cigarette smoking, frequency and duration; BMI; education level; energy, fruits, vegetable, fish intake. | 8 | >3 |
| Freedman ND et al. | 2007 | USA | | | ≧50 | 14-15 | 474,606 | ≧39 g/d vs. 0 | 1.57（0.98-2.52） | Age; fruit, vegetable, total energy intake; categorical variables for sex, BMI, education level. | 8 | >3 |
| Sung NY et al. | 2007 | Korea | | | ≧30 | 6.5 | 669,570 | ≧25 g/d vs. 0 | 1.2  (1.1-1.4) | Age, BMI, alcohol intake, preference for salty food. | 7 | >3 |
| Larsson SC et al. | 2006 | Sweden | | | 49-83 | 7-18 | 66,651 | ≧5.71 g/d vs. 0 | 1.33 (0.79-2.25) | Age; education level; fruit, vegetable, processed meat intake. | 7 | >3 |
| Sjodahl K et al. | 2006 | Norway | | | ≧15 | 18 | 69,962 | 5 times/14 days vs. 0 | 1.49 (0.78-2.83) | Sex, education, BMI, tobacco smoking. | 7 | >3 |
| Nakaya N et al. | 2005 | Japan | | | 40–64 | 7 | 21,201 | ≧45.6 g/d vs. 0 | 1.0  (0.7-1.4) | Age; cigarette smoking; education level; daily consumption of orange, other fruits, juice, spinach, carrot or pumpkin, tomato. | 7 | >3 |
| Barstad B et al. | 2005 | Denmark | | | 21–93 | 28 | 28,463 | High vs. low | 1.13 (0.41-1.86) | Age; sex; education level; smoking; inhalation; BMI; physical activity; total alcohol and beer, wine, spirits intake. | 6 | >3 |
| Fujino Y et al. | 2002 | Japan | | | ≧18 | 2 | 127,477 | Current vs. Never | 0.72 (0.5-1.05) | Age, dietary choices (including pickles, vegetables, fruit, green tea, preference for salty foods). | 7 | <3 |
| Sasazuki S et al. | 2002 | Japan | | | 40–59 | 10 | 19,657 | 322.5 g/week vs. 0–3 days/month | 1.1  (0.8-1.6) | Age; area of residence; smoking habit; alcohol consumption; consumption of fruit, green or yellow vegetables, salted cod roe or fish gut; BMI. | 8 | >3 |
| Galanis DJ et al. | 1998 | USA | | | ≧18 | 14.8 | 11,907 | ≧3 times/day vs. 0 | 0.6  (0.3-1.2) | Age, years of education, Japanese place of birth, smoking or alcohol consumption status. | 8 | >3 |
| Kato I et al. | 1992 | Japan | | | Male ≧ 40 y; Female≧ 30 y | 6 | 9,753 | ≧50 ml/day vs. 0 | 2.75 (1.2-6.29) | Smoking, cooking methods, family history of stomach cancer, age, sex. | 7 | <3 |
| Stemmermann GN et al. | 1990 | USA | | | ≧45 | 8 | 8,006 | ≧40 oz/month vs. 0 | 1.17 (0.73-1.90) | Age, current smoker status, age started smoking, number of cigarettes smoked per day, ex-smoker status, maximum number of cigarettes smoked. | 7 | <3 |
| Kono S et al. | 1987 | Japan | | | 27-89 | 19 | 5,130 | ≧54 ml/day vs. 0 | 1.17 (0.66-2.07) | Age, smoking status. | 6 | <3 |

USA = United States of America; BMI = body mass index; RR = relative risk; CI = confidence interval; IF = impact factor; COPD = chronic obstructive pulmonary disease; DM = diabetes mellitus; oz = ounces; NOS: Newcastle–Ottawa Quality Assessment Scale.
